# Evaluation of allograft contamination and decontamination at the Treviso Tissue Bank Foundation: A retrospective study of 11,129 tissues

**DOI:** 10.1371/journal.pone.0173154

**Published:** 2017-03-07

**Authors:** Adolfo Paolin, Diletta Trojan, Pieter Petit, Paola Coato, Roberto Rigoli

**Affiliations:** 1 Treviso Tissue Bank Foundation, Treviso, Italy; 2 Foundation European Tissue Banks, Berlin, Germany; 3 Department of Clinical Pathology, Regional Hospital, Treviso, Italy; FDA, UNITED STATES

## Abstract

Microbiological contamination of retrieved tissues has become a very important topic and a critical aspect in the safety of allografts. We have analysed contamination in 11,129 tissues with a longitudinal contamination profile for each individual tissue. More specifically, 10,035 musculoskeletal tissues and 1,094 cardiovascular tissues were retrieved from a total of 763 multi-tissue donors, of whom 105 were heart-beating donors as well as organ donors, while the remaining 658 were non-heart beating donors and tissue donors only. All tissues were decontaminated twice, the first time immediately after retrieval and the second time after processing. Each tissue was submitted to microbiological culture three times, i.e., upon retrieval (Time 1), after the first decontamination (Time 2) and after the second decontamination (Time 3). The contamination rate for musculoskeletal tissues was 52%, 16.2% and 0.5% at Time 1, 2 and 3, respectively. The contamination rate for cardiovascular tissues was 84%, 42% and 6%. More than one strain was simultaneously present in 10.8% of musculoskeletal tissues and 44.6% of cardiovascular tissues. Out of 8,560 non-heart-beating donor musculoskeletal tissues, 4,689 (54.8%), 1,383 (16.2%) and 42 (0.5%) were contaminated at Time 1, Time 2 and Time 3, respectively. Out of 1,475 heart-beating donor musculoskeletal tissues, 522 (35.4%) 113 (7.7%) and 2 (0.1%) tissues were found to be contaminated at Time 1, 2 and 3, respectively. Out of 984 non-heart beating donor cardiovascular tissues, 869 (88.3%), 449 (45.6%) and 69 (7%) proved positive at Time 1, 2 and 3 respectively, while 50 (45.5%) and 10 (9.1%) heart-beating donor cardiovascular tissues were contaminated at Time 1 and 2. No tissue was contaminated at Time 3. Based on our methods, the two-step decontamination approach is mandatory in order to drastically reduce the number of tissues found to be positive at the end of the process.

## Introduction

Donated tissues from cadaveric donors are successfully used in orthopaedic, maxillofacial, cardio-vascular, plastic surgery and other medical specialties. Microbiological contamination of retrieved tissues has become a very important topic and it is a critical aspect in the safety of allografts, especially from multi-tissue donors whose tissues are frequently contaminated as a consequence of the retrieval and handling process. As contaminated tissues may represent a potential risk for recipients [1,2] every tissue bank adopts specific decontamination procedures. Grafts are sterilized by means of antibiotic (AB) cocktails [3–5], and terminal irradiation although the latter may lead to mechanical functional losses and alter the biological properties of the tissues [6,7]. However, bacterial contamination remains one of the major causes for discarding tissues, with spectra and rates varying widely in relation to the type of donor and tissue. Cardiac tissues are usually more contaminated than musculoskeletal tissues [8] and skin commensals such as Coagulase Negative Staphylococcus (CNS) are the most commonly isolated organisms [9–11].

Aiming at defining the best decontamination protocol, De By et al. analysed in 2012 the decontamination methods used in 17 European cardiovascular tissue banks and found major methodological differences among the various laboratories, highlighting the need to validate and standardize the procedures [12]. The differences between the protocols lie in the composition and concentration of the decontaminating solutions as well as the duration and temperature of incubation. Despite the antiseptic measures adopted in all the processing phases, a high percentage of tissues still remain contaminated, thus preventing their clinical use. The goal of every tissue bank is to achieve germ-free allografts after decontamination and the choice of the correct composition and the modalities of use of the AB cocktail are crucial. An extensive retrospective analysis of our contamination data for all tissue types was carried out with the purpose of validating a more effective decontamination procedure. In particular, the results of microbiological cultures carried out on retrieved tissues over a period of 4 years were submitted to a longitudinal analysis in order to evaluate the tissue contamination rate and the decontaminating efficacy of the AB cocktail which is currently used at our tissue bank.

## Materials and methods

### Data collection

Bacterial contamination was analysed in 11,129 consecutive tissues that were retrieved, processed and stored by Treviso Tissue Bank Foundation (FBTV). A longitudinal contamination profile was defined for each individual tissue. Accordingly, we excluded those tissues that had been discarded post-retrieval as unsuitable for clinical use due to morphological abnormalities or because the donor was positive for one of the relevant serological markers. The overall discard rate for the aforementioned causes was 42% for cardiovascular tissues (CVT) and 7% for musculoskeletal tissues (MST). The tissues included in this survey were 10% CVT and 90% MST, accounting for 58% and 93% of all retrieved CVT and MST, respectively. More specifically we have analysed 10,035 MST, and 1,094 CVT (heart valves, pericardium, arteries and veins) retrieved from a total of 763 multi-tissue donors. Of these, 105 heart-beating donors (HBD) were also organ donors, while the remaining 658 non-heart beating donors (NHBD) were tissue donors only. Our retrieval team of physicians and technicians harvested the tissues in the operating theatre after organ retrieval from HBD, and within 24h of cardiac arrest from NHBD. Prior to tissue retrieval the skin underwent surgical scrubbing with chlorhexidine solution and shaving followed by an additional application of chlorhexidine and povidone iodine. The tissues were then brought to the bank and processed in biohazard class-II laminar airflow cabinets, in a class B environment facility.

## Decontamination method

All tissues were decontaminated twice, the first time immediately after retrieval and the second time after processing, with an AB cocktail of Ceftazidime 240μg/ml (Fresenius-Kabi), Lincomycin 120μg/ml, Polymyxin B 100μg/ml (Biochrom) and Vancomycin 50μg/ml (Hospira). The tissues were kept in RPMI medium with the AB cocktail at +4°C for 24-48h each time. This procedure of decontamination was the same as that in force at the European Homograft Bank [3] when it was adopted by FBTV, except for Cefoxitin which was replaced in our AB cocktail by Ceftazidime.

## Microbiological analysis

Microbiological cultures for aerobic and anaerobic bacteria, fungi/yeasts and mycobacteria were carried out three times for each tissue, i.e. upon retrieval (Time 1), after the first decontamination (Time 2) and after the second decontamination (Time 3). Microbiological cultures were carried out using BD BACTEC^™^/Alert Fluorescent Test Technology plus aerobic/F Medium and anaerobic/F culture vials, Soybean-Casein Digest Broth (BD, Becton, Dickinson and Company, New Jersey). Culture bottles were incubated at 36.5°C for 7 days. Each vial contained a chemical sensor to detect increases in CO_2_ produced by the growth of microorganisms and fluorescence, which was subsequently monitored by a BACTEC/Alert fluorescent series instrument. Culture bottles showing evidence of growth after 7 days were gram stained, sub-cultured on blood agar plates and incubated for 48h at 35/38°C in a normal atmosphere, a 5% CO_2_-enriched atmosphere, and an anaerobic atmosphere. The microorganisms in the test sample inoculated in the BACTEC vial metabolize the substrates producing CO_2_. The increased fluorescence caused by higher amounts of CO_2_ is detected by the BACTEC fluorescent series instrument. The analysis of the rate and amount of CO_2_ increase enables the BACTEC fluorescent series instrument to determine if the vial is positive, i.e., if the test sample contains viable organisms. Samples were then processed under a biohazard class-II laminar flow hood and all bacteria were identified with the standard biochemical procedure. Lastly, an antibiogram was drawn up for each bacterium isolated and the Minimal Inhibitory Concentration estimated in the standard media used in clinical practice. Samples were also cultured in a Lowenstein-Jensen medium to isolate mycobacteria. The bacteriological examination was performed 3 times: on the samples of the first isotonic solution (8–10 ml) in which the tissues were rinsed upon retrieval, and after each of the two decontamination steps in order to minimize the risk of AB carry-over. The rinsing solution of each tissue was sampled without filtering and all procedures were carried out at room temperature. Microbiological cultures and analyses were carried out by an accredited in-hospital microbiology laboratory and interpreted by a microbiologist with specific expertise. In compliance with our policy, the following strains were classified as non-compliers: *Clostridium spp*., *Fungi/Yeast*, *Mycobacteria*, *Streptococcus pyogenes*, *Streptococcus pneumoniae*, *Pseudomonas aeruginosa*, *Serratia marcescens*, *and Meningococcus*. Whenever any of these strains were isolated the tissue was discarded regardless of the step at which positivity was detected. In addition to discarding tissues contaminated with non-compliers, all tissues found to be positive after the 2nd decontamination were also discarded.

## Results

### MST contamination

MST microbiological findings are summarised in Table 1. Out of 10,035 MST, 5,211 (52%) were contaminated at Time 1, while 1,496 (15%) and 44 (0.4%) proved positive at Time 2 and Time 3, respectively. *Coagulase Negative Staphylococci* (*CNS*) were the most commonly isolated microorganisms at Time 1 (82.6% of the positive cultures) followed by *Streptococcus spp*., *Bacillus spp*., *Clostridium spp*. and *Staphylococcus aureus*, which disappeared almost totally at Time 3 after the two following decontamination steps.

**Table 1:** Percentage of contamination and bacteria found at Times 1, 2 and 3 in MST.

Time 1			Time 2			Time 3		
Microorganism	N° of tissues	%	Microorganism	N° of tissues	%	Microorganism	N° of tissues	%
*Coagulase -Staphylococcus*	**4,304**	**82.6**	*Coagulase -Staphylococcus*	**1,217**	**81.4**	*Coagulase -Staphylococcus*	**21**	**47.7**
*Staphylococcus aureus*	**91**	**1.7**	*Staphylococcus aureus*	**35**	**2.3**	-	**-**	**-**
*Streptococcus* spp	**252**	**4.8**	*Streptococcus* spp	**45**	**3.0**	-	**-**	**-**
*Bacillus* spp	**145**	**2.8**	*Bacillus* spp	**69**	**4.6**	-	**-**	**-**
*Clostridium* spp	**142**	**2.7**	*Clostridium* spp	**7**	**0.5**	*Clostridium* spp	**5**	**11.4**
*Escherichia* spp	**55**	**1.1**	*Escherichia* spp	**6**	**0.4**	-	**-**	**-**
*Enterococcus* spp	**52**	**1.0**	*Enterococcus* spp	**42**	**2.8**	*Enterococcus* spp	**10**	**22.7**
*Others (27 genera)*	**170**	**3.3**	*Others (14 genera)*	**75**	**5.0**	*Others (4 genera)*	**8**	**18.2**
**Total tissues**	**5,211 (52%)**		**Total tissues**	**1,496 (15%)**		**Total tissues**	**44 (0.4%)**	
**Total MST analysed 10.035**								

#### NHBD vs HBD in MST

Tables 2–4 show the microbiological data for the NHBD and HBD groups.

**Table 2:** N° of tissues contaminated and contamination rate in MST of HBD and NHBD at Times 1, 2 and 3.

	Tissues analysed	Time 1		Time 2		Time 3	
		n°	%	n°	%	n°	%
**NHBD** MST	8,560	4,689	54.8	1,383	16.2	42	0.5
**HBD** MST	1,475	522	35.4	113	7.7	2	0.1
Total MST	**10,035**	**5,211**		**1,496**		**44**	

**Table 3:** Number of bacterial isolates/number of tissues in MST of HBD and NHBD at Times 1, 2 and 3.

	Time 1		Time 2		Time 3	
	NHBD	HBD	NHBD	HBD	NHBD	HBD
**n° of strains/n° of tissues**	5,274 /4,689	544/522	1,527/1,383	120/113	42/42	2/2

**Table 4:** Number and percentage of bacterial isolates per tissue in MST of HB and NHBD at Times 1, 2 and 3.

	Time 1		Time 2		Time 3	
Contaminated tissues	NHBD n° = 4,689	HBD n° = 522	NHBD n° = 1,383	HBD n° = 113	NHBD n° = 42	HBD n° = 2
	%	%	%	%	%	%
One strain	89.2	96.2	91.5	93.8	100	100
Two strains	9.4	3.4	6.5	6.2	-	-
More than two strains	1.4	0.4	2.0	-	-	-
Total MST	**5,211**		**1,496**		**44**	

In the NHBD group, out of 8,560 MST, 4,689 (54.8%), 1,383 (16.2%) and 42 (0.5%) were contaminated at Time 1, Time 2 and Time 3, respectively, with a total of 5,274, 1,527 and 42 strains isolated at the same Time points. Approximately 90% of MST from NHBD were contaminated by a single strain at Time 1 and 2, and 100% at Time 3 and approximately 10% of MST were contaminated by more than one strain at Time 1 and 2.

In the HBD group, out of 1,475 MST, 522 (35.4%) 113 (7.7%) and 2 (0.1%) were contaminated at Time 1, 2 and 3, respectively, with a total of 544, 120 and 2 strains isolated at the same Time points. Almost all MST were contaminated by a single strain at any Time, with a few tissues (<7%) contaminated by more than one strain at Time 1 and 2.

### CVT contamination

CVT microbiological findings are summarised in Table 5. Out of 1,094 CVT, 919 (84%) were contaminated at Time 1, while 459 (42%) and 69 (6%) proved positive at Time 2 and Time 3, respectively. *CNS* was the most commonly isolated microorganism at Time 1 (46.6% of positive cultures) followed by *Streptococcus spp*., *Clostridium spp*, *Staphylococcus aureus* and *Escherichia spp*., which were not completely eradicated by the two decontaminations.

**Table 5:** Percentage of contamination and bacteria found at Times 1, 2 and 3 in CVT.

Time 1			Time 2			Time 3		
Microorganism	N° of tissues	%	Microorganism	N° of tissues	%	Microorganism	N° of tissues	%
*Coagulase—Staphylococcus*	428	46.6	*Coagulase Staphylococcus*	184	40.1	*Coagulase—Staphylococcus*	3	4.3
*Staphylococcus aureus*	33	3.6	*Staphylococcus aureus*	19	4.1	*Staphylococcus aureus*	3	4.3
*Streptococcus spp*.	176	19.2	*Streptococcus spp*.	116	25.3	*Streptococcus spp*.	12	17.4
*Clostridium spp*.	78	8.5	*Clostridium spp*.	32	7.0	*Clostridium spp*.	5	7.2
*Escherichia spp*.	31	3.4	*Escherichia spp*.	1	0.2	-	-	-
*Klebsiella spp*.	23	2.4	-	-	-	-	-	-
*Enterococcus spp*.	21	2.3	*Enterococcus spp*.	20	4.4	*Enterococcus spp*.	18	26.1
Others (22 genera)	129	14.0	Others (18 genera)	87	18.9	Others (8 genera)	28	40.7
**Total tissues**	**919 (84%)**		**Total tissues**	**459 (42%)**		**Total tissues**	**69 (6%)**	
Total CVT analysed 1,094								

#### NHBD vs HBD in CVT

Tables 6–8 show the microbiological data of the NHBD and HBD groups.

**Table 6:** N° of tissues contaminated and contamination rate in CVT of HBD and NHBD at Times 1, 2 and 3.

	Tissue analysed	Time 1		Time 2		Time 3	
		n°	%	n°	%	n°	%
**NHBD** CVT	984	869	88.3	449	45.6	69	7.0
**HBD** CVT	110	50	45.5	10	9.1	-	-
Total CVT	**1,094**	**919**		**459**		**69**	

**Table 7:** Number of bacterial isolates/number of tissues in CVT of HBD and NHBD at Times 1, 2 and 3.

	Time 1		Time 2		Time 3	
	NHBD	HBD	NHBD	HBD	NHBD	HBD
**n° of strains / n° of tissues**	1,389/869	60/50	615/449	10/10	69/69	-

**Table 8:** Number and percentage of bacterial isolates per tissue in CVT of HBD and NHBD at Times 1, 2 and 3.

	Time 1		Time 2		Time 3	
	NHBD n° = 869	HBD n° = 50	NHBD n° = 449	HBD n° = 10	NHBD n° = 69	HBD n° = 0
**Contaminated tissues**	**%**	**%**	**%**	**%**	**%**	**%**
One strain	55.4	84	70.4	100	100	-
Two strains	31.6	14	22.9	-	-	-
More than two strains	13.0	2	6.7	-	-	-
Total CVT	**919**		**459**		**69**	

In the NHBD group, out of 984 CVT 869 (88.3%), 449 (45.6%) and 69 (7%) proved positive at Time 1, 2 and 3, respectively, with a total of 1,389, 615, and 69 strains isolated at the same Time points. In this group, 55.4% and 44.6% of CVT were contaminated at Time 1 by single and multiple strains respectively, while 70.4% and 29.6% of CVT were contaminated at Time 2 by single and multiple strains respectively. At Time 3 only single strain contamination was found.

In the HBD group, 50 (45.5%) and 10 (9.1%) CVT were contaminated at Time 1 and 2, with a total of 60 and 10 strains isolated at the same Time points. No tissues were contaminated at Time 3. In the entire HBD group, 84% and 100% of CVT were contaminated by a single strain at Time 1 and Time 2, respectively. No contamination by multiple strains was detected at Time 2 and Time 3.

## Discussion

We have carried out a comprehensive analysis of the frequency and genera of bacteria isolated in allografts from cadaveric donors in a multi-tissue bank during 4 consecutive years. The overall contamination rate at retrieval was 52%, in MST, and 84% in CVT. More than one strain per tissue was isolated in 10.8% of MST and 44.6% of CVT, with a mean ratio of 1.1 strains per tissue in MST and 1.6 in CVT. Our data show that MST and CVT from NHBD show a higher degree of contamination than tissues from HBD. Moreover, MST are contaminated predominantly by one single strain at each Time point in both groups, and multiple strains are detected in only about 10% of the tissues. CVT from NHBD were contaminated to a higher extent and with a higher incidence of multiple strains than CVT from HBD. Lastly, MST and CVT from NHBD still show high positivity at Time 3, after the two decontamination steps. *CNS* was the most frequently isolated low-pathogenic strain and *Streptococcus spp*. was the most frequent highly pathogenic strain in both MST and CVT. This finding is comparable with the results of other studies [8,9,11,13–15]. The most notable difference between the two types of tissues was that CVT showed a higher percentage of contamination by germs belonging to intestinal and upper airways flora. Deijkers et al. reported graft contamination by low pathogenic microorganisms in 50%, and by highly pathogenic microorganisms in 3% of MST. Vehmeyer et al. found a contamination rate of 45% in allografts retrieved from cadaveric bone donors, while Ibrahim et al. found a contamination rate of 27% [9,11]. The CVT contamination rates resulting from our analysis are comparable to those reported by Tabaku et al. [10] in their sample of 948 CVT from 491 donors who, unlike ours, were primarily HBD.

*Contamination by CNS—*mostly skin commensals—is probably caused by external contamination at the time of procurement due to a leakage from the skin incisions made to access the thoracic and abdominal cavities, exposure to the environment during retrieval, and handling. The method used to culture the specimen can amplify the presence of one strain to many colonies, as suggested by Ibrahim [15]. Conversely, the high percentage of bacterial strains from intestinal and upper airways flora in CVT may be due to multiple causes. Our donors were mainly NHBD whose cause of death was traumatic (55%) or cardiac (fatal heart attack, 42%). Traumatic causes exponentially increase the risk of bacterial contamination as confirmed by Deijkers et al. who report that the risk of graft contamination with highly pathogenic organisms is increased by a factor of 3.4 after a traumatic cause of death [9]. The same explanation is also given by Martinez et al. who argue that seeding of the blood-stream could develop in victims of extensive trauma or following invasive procedures during the emergency period such as in our donors, who were victims of road accidents and heart attacks [16].

CVT retrieval from the thoracic and abdominal cavities of donors who often present with trauma-induced haemorrhagic effusion can facilitate passive and active cross-contamination. Moreover, NHBD are often transferred from the site of death to the hospital hours after cardiac arrest, with a prolonged warm ischemic time that might favour the growth and migration of bacteria into the blood prior to body refrigeration in the morgues of the referring hospital. HBD tissues were retrieved immediately after the removal of organs, only a few hours after circulatory arrest, whereas NHBD tissues were retrieved on average 17 hours after the circulation stopped. Van Kats et al. also confirmed a significant relationship between warm ischemic time and contamination at retrieval [17]. Lastly, when our retrieval team collects all the tissues that donors are potentially suitable to provide, retrieving multiple tissue types takes time, with the retrieval process requiring a team of at least 4–5 people. All these variables increase the risk of tissue contamination at retrieval as evidenced by Lannau et al. in a sample of 281 cadaveric donors [18].

Our analysis has revealed that in the whole sample considered, 68% of tissues that were positive at retrieval became negative after the first decontamination, and a further 30% became negative after the second decontamination, while 2% remained positive until the end. Both decontamination steps were most effective in MST, with a final residual positivity of only 0.4%. Conversely, both decontamination steps of CVT were remarkably less effective, with 6% of CVT remaining positive after the second decontamination. However, while contamination in MST was mainly attributed to skin commensals, in CVT it was largely due to potentially pathogenic germs of which several strains were detected concurrently. Our overall decontamination data are in line with the findings reported by Tabaku et al. in 2004: their CVT sample showed a decontamination rate of 82.5% after the first decontamination and a final sterility rate of 94% [10]. Ireland and Spelman showed a contamination rate of 0.5% for MST after AB treatment [8]. Hence, the decontamination method chosen may have a significant impact on the contamination rate. Decontamination protocols differ widely among tissue banks as regards the type and concentration of ABs used as well as the temperature and duration of exposure of tissues to the AB cocktail. The specific protocol for decontamination we have adopted from European Homograft Bank [3] proved to be effective against a wide spectrum of microorganisms isolated from allografts specimens, without having any harmful effect on the allograft structure. It is worth noting that in spite of the two decontaminations steps there were still some positive microbial cultures at the end of the process and, therefore, the number of tissues discarded for microbiological reasons has always been high, as reported by other authors as well. Several papers address the topic of allograft contamination, in CVT [12, 19–21], MST [9,11,22] and multi-tissue banks [8], but their results are difficult to compare as they adopt different standards for microbiological screening as well as for the decontamination of tissues. The rate of positive cultures may be influenced by the sensitivity of the bacterial culture assay used to evaluate tissue contamination. For instance, we introduced the BACTEC method as the standard for microbiological cultures instead of the previous BHI (Brain Heart Infusion). This caused a major increase in positivity for all types of strains, including gram-negative and sporogenous bacteriae. Our overall decontamination rate in CVT after the first decontamination is comparable to that of previous reports [14], although our sample was mainly comprised of NHBD. There might be several explanations for the residual percentage of contaminated tissues at the end of the process. Multiple contaminations, found almost exclusively in CVT, might explain the incomplete decontamination achieved in this type of tissue, a hypothesis supported by the finding that MST, contaminated in 90% of cases by a single strain, were virtually completely decontaminated at the end of the process.

Another explanation could be that the AB cocktail may be less effective at low temperatures as demonstrated by Germain et al. in their study on heart tissues, showing a slight decrease in bacterial contamination after decontamination at 4°C [23]. In contrast, Fan et al. reported that an AB cocktail of lincomycin, polymyxin B and vancomycin reduced the bacterial contamination rate by 76.8% at 4°C for at least 20h [14]. However, two-step decontamination procedure was effective even at low temperatures in most tissues, including CVT, suggesting that the initial bio-burden was probably very low. This was proved by Germain et al. who found very low numbers of CFU/ml in the heart valve transport medium for both aerobic and anaerobic strains [23]. Unlike the above-mentioned study, our laboratory carried out a qualitative assessment of the microbiological results. Therefore, CFU’s were not quantified in the positive cultures. The contamination level might also be underestimated in the case of slow growing microorganisms such as *Propionibacterium* and *Corynebacterium* which require longer incubation times than those used to detect other bacterial strains [17]. This may partly explain the low rate of decontamination of these specific strains, as AB is known to be effective only when the bacterium present in tissue is actively replicating [19].

False negative cultures may also occur after using ABs, as suggested by Buzzi et al. and Gatto et al.. This is due to traces of AB remaining in the tissue after decontamination [24,25]. In our case, tissues were rinsed with isotonic saline at every step of the microbiological control after removing them from the AB solution. It may be assumed that the concentration of residual AB in the rinsing solution was very low and in any event too low to inhibit bacterial growth given the remarkable percentage of positive tissues detected in our sample post-decontamination. At any rate the question remains as to whether it is advisable to perform decontamination and microbiological monitoring in two steps rather than in one single step at the end of processing. In our analysis, the first decontamination was very effective in those MST that were contaminated by one single strain. Conversely, it was less effective in those CVT which harboured multiple contaminations corresponding to almost 50% of cases. Consequently, the two-step decontamination approach, based on our methods, appears crucial in drastically reducing positive outcomes at the end of the process.

Underestimating the microbiological risk can cause serious adverse events such as those described in the literature, with a share of infections in recipients caused by allografts [26] contaminated with highly pathogenic germs. Therefore, based on our experience we recommend to carry out microbiological controls at every stage of the process, using sensitive tests capable of effectively detecting contamination. Targeted decontamination minimises the risk of false negatives and, consequently, the risk of infections in recipients. Lastly, considering the importance of bacteria in allografts we are currently developing and validating a new and more effective AB cocktail with different exposure times and temperatures in order to further reduce the overall bio-burden and the waste of donated tissues.
